# Editorial: Metabolic dysregulation in hematological malignancies: emerging insights and therapeutic implications

**DOI:** 10.3389/fonc.2025.1701952

**Published:** 2025-10-30

**Authors:** Pengcheng Liu, Jiao Ma, Zizhen Xu

**Affiliations:** ^1^ Department of Laboratory Medicine, College of Health Science and Technology, Ruijin Hospital, Shanghai Jiao Tong University School of Medicine, Shanghai, China; ^2^ Department of Biochemistry and Molecular Cell Biology, Shanghai Jiao Tong University School of Medicine, Shanghai, China

**Keywords:** leukemia, lymphoma, hematological malignancies, metabolic dysregulation, prognosis, target therapy

There is growing evidence that highlights the critical role of metabolic reprogramming in the development and progression of hematological malignancies. Cancer cells alter metabolic pathways—such as enhanced glucose uptake (the Warburg effect), glutamine metabolism, and fatty acid synthesis—to fuel rapid proliferation and adapt to the tumor microenvironment. These changes not only supply energy and biomolecules but also promote drug resistance and disease progression. Understanding these metabolic aberrations may unveil novel therapeutic targets for more precise and effective treatments.

This Research Topic published five articles covering the broad spectrum of basic and clinical research on macromolecular metabolism in Hematological Malignancies.

## Mitochondrial gene signature in DLBCL prognosis

Wang et al. developed a prognostic model based on nine mitochondria-related genes that effectively stratifies patients with Diffuse Large B-cell Lymphoma (DLBCL). The model demonstrated strong predictive accuracy (AUC >0.6, exceeding 0.7 in some cohorts), enabling personalized treatment approaches. Functional studies on PCK2, a gene in the model, revealed that its knockdown suppressed DLBCL cell proliferation under low glucose conditions, suggesting its potential as a therapeutic target.

## Lipid metabolism in multiple myeloma

In a review by Wang et al., MM cells were shown to depend heavily on *de novo* fatty acid synthesis and bone marrow adipocytes for lipid supply. Malignancies tend to manipulate lipid metabolism to facilitate their growth and alter the surrounding environmen (1), and in this case, Disrupted lipid metabolism was found to foster an immunosuppressive microenvironment involving lipid-rich tumor-associated macrophages, though the precise mechanisms remainunclear.

## Chiral metabolome analysis in pediatric BCP-ALL

Collins et al. identified a distinct chiral metabolic phenotype in patients with IgH-aberration-positive B-cell precursor acute lymphoblastic leukemia (BCP-ALL). Notably, D-amino acids were detected in leukemic cells for the first time, and chiral metabolic signatures correlated with treatment resistance. These signatures could serve as potential diagnostic and prognostic biomarkers, and the interplay between chiral metabolomes and their corresponding chiral enzymes and drugs could be future focus (2).

## Nutritional status and MM outcomes

Jin et al. conducted a meta-analysis (9 studies, 1,176 patients) linking high Controlling Nutritional Status (CONUT) scores with worse overall survival in MM (HR = 1.87, *p* < 0.001), although no significant association was found with progression-free survival. Their findings underscore the importance of nutritional assessment in MM management.

## Hyperuricemia and hyperuricosuria in lymphoma/MPN

Kunlayawutipong et al. reported high prevalence rates of hyperuricemia (43.6%) and hyperuricosuria (39.4%) in patients with lymphoma and myeloproliferative neoplasms (MPNs). Key risk factors included: Lymphoma: Reduced kidney function (eGFR <90) and elevated LDH (≥250 U/L) for hyperuricemia; high LDH for hyperuricosuria. MPN: Hemoglobin <10 g/dL and LDH ≥640 U/L predicted hyperuricosuria. These insights could guide uric acid-lowering therapies to prevent complications.

## Conclusion

Metabolic research is uncovering pivotal biomarkers and therapeutic vulnerabilities in hematologic malignancies (1, 3). From mitochondrial gene signatures to lipid metabolism and nutritional influences, these advances pave the way for innovative, targeted treatments. We hope this synthesis of recent findings provides valuable insights for researchers and clinicians alike.
